# Prescription of off-label medications in patients on dialysis: time to challenge contraindications?

**DOI:** 10.1093/ckj/sfaf319

**Published:** 2025-10-27

**Authors:** Maxime Touzot, Sonia Amato, Christophe Ridel, Pablo Ureña-Torres

**Affiliations:** Owkin, Biomedical department, Paris, France; Aura Paris Plaisance, Paris, France; FME Global Medical Office, Fresnes, France; AURA Nord Saint Ouen, Saint Ouen, Francia and Department of Renal Physiology, Necker Hospital, University of Paris Descartes, Paris, France

**Keywords:** clinical trial, dialysis, drug, off-label, pharmacokinetics

## Abstract

Prescribing off-label medications for patients undergoing dialysis presents significant clinical challenges that require careful judgement to optimize the risk–benefit ratio. The altered pharmacokinetic profile in this population, characterized by impaired renal elimination, modified non-renal clearance pathways, and complex polypharmacy, has traditionally led to numerous contraindications, not solely due to concerns about drug accumulation but also to prevent serious adverse events. While established dosing protocols exist for certain drug classes, robust pharmacological data remain insufficient for many contemporary medications, leaving clinicians without evidence-based guidance for commonly prescribed authorized therapies in participants without significant chronic renal disease. The categorical off-label medication contraindications in haemodialysis warrants new reconsideration in the light of emerging evidence that has successfully challenged these constraints through well-designed clinical studies and real-world experiences. In this review, we aim to examine the evidence on selected examples of off-label medications previously deemed contraindicated that have been successfully evaluated in patients treated by dialysis, and explore emerging therapeutic agents. Finally, we discuss the clinical, research, methodological, and regulatory barriers that must be addressed to improve evidence-based prescribing in patients on dialysis.

## INTRODUCTION

Prescribing off-label medications for patients with end-stage renal disease (ESRD), especially those undergoing dialysis, is challenging as it requires careful clinical judgement to optimize the risk:benefit ratio. Owing to altered pharmacokinetics, including impaired renal elimination and changes in drug metabolism, many drugs are contraindicated to prevent serious adverse events. In some cases, dose adjustments are often necessary. In addition, polypharmacy, common in this population, requires rigorous and systematic evaluation of potential drug–drug interactions. While established dosing protocols exist for certain drug classes such as antibiotics, antifungals, antivirals, and chemotherapy agents, robust pharmacological data remain lacking for many recent medications. This is largely due to the systematic exclusion of patients with ESRD from pivotal clinical trials, leaving clinicians without evidence-based guidance for many commonly used drugs in participants without significant chronic kidney disease (CKD).

This gap in knowledge creates a clinical dilemma; either discontinuing effective therapies when a patient initiates dialysis or withholding potentially beneficial drugs solely due to regulatory constraints. Although many clinicians and pharmacists are deterred by theoretical contraindications, some have moved beyond these barriers, initiating treatment in small patient cohorts with promising outcomes [1–5]. These real-world experiences can pave the way for larger observational studies and, eventually, randomized controlled/clinical trials (RCTs) to validate proof-of-concept [6]. A notable example is the use of direct-acting oral anticoagulants (DOACs) in patients on dialysis. Despite early theoretical concerns, recent well-designed RCTs have demonstrated both the safety and efficacy of these agents compared with traditional oral anticoagulants [6]. In this paper, we review selected examples of drugs previously considered contraindicated but now successfully tested in patients receiving dialysis therapy. We focus mainly on non-oncologic drugs and emerging therapies where evidence is lacking. Finally, we discuss potential applicability in this population, and the clinical and regulatory barriers that should be addressed in the future.

### Drugs contraindicated in patients on dialysis: from theory to reality

#### Pharmacokinetics in patients on dialysis

As the kidneys represent a major organ for the drug elimination, renal impairment alters the pharmacokinetics and consequently, systemic drug exposure. Reduce renal clearance requires a dosage adjustment (dose reduction and/or longer dosing interval) to prevent systemic accumulation and toxicity of active metabolites. Although renal toxicity itself is no longer a concern in patients on dialysis (except for preserving residual diuresis), adverse effects remain the major concern. Patients on dialysis have a unique and complex pharmacotherapeutic profile, resulting from altered absorption, distribution and non-renal metabolism and drug-transport [7–11]. These changes include:

- a decrease in plasma protein binding (hypoalbuminaemia, competition by uraemic toxins), increasing the free active fraction of certain drugs;
- a reduction in tissue binding and an increase in the distribution volume of water-soluble molecules, promoting accumulation in the extracellular compartment;
- alterations in gastrointestinal absorption due to delayed gastric emptying, higher gastric pH, or changes in the intestinal microbiota, which may alter the bioavailability of certain oral treatments;
- a decrease in hepatic and intestinal metabolism (reduced activity of certain cytochrome P450 isoforms, slowed glucuronidation), which may potentiate the toxicity of molecules.

**Figure 1:**
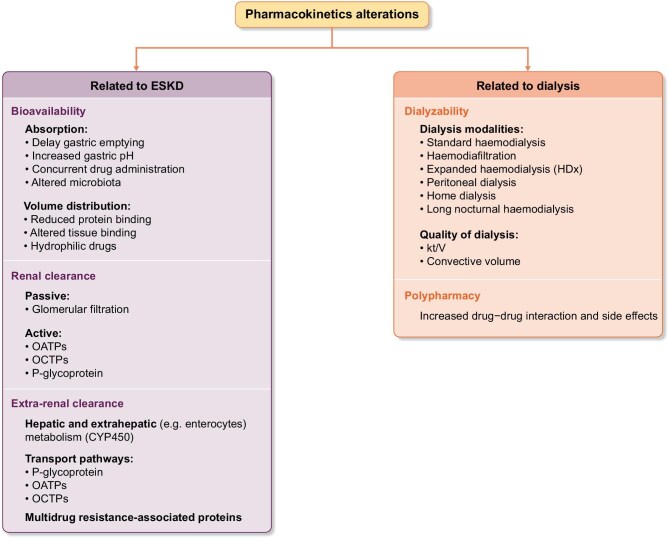
Consideration before off-label use of drugs in patients under dialysis therapy. Abbreviations: OATPs, organic anion-transporting polypeptides; OCTPs organic cation-transporting polypeptides.

All of them contribute to unpredictable drug responses. (Figure 1) [8, 9]. Numerous comprehensive and critical reviews of these topics have been published previously [8–11].

The intermittent nature of dialysis therapy furthers adds variability to drug exposure as it may potentially remove therapeutic agents [12]. Drug interactions and nutritional status also affect bioavailability in various ways [13], especially for drugs with narrow therapeutic windows, high renal clearance, or hydrophilic properties [12]. Understanding drug’s dialysability is then key to optimize dosing: either delaying dosing until post-dialysis time to maintain efficacy or, conversely, using dialysis to enhance tolerance by removing the excess drug. Finally, polypharmacy is common in this population, often >8–10 medications daily, increases the risk of drug–drug interactions and adverse effects [14–16].

Many agents are labelled ‘not recommended’ in patients on dialysis, primarily due to toxicity concerns, although robust clinical evidence is often limited [17, 18]. Emerging pharmacokinetic models and clinical data suggest that with careful monitoring and individualized dose adjustments, some previously contraindicated drugs may be reconsidered in selected cases [8, 10, 19, 20].

#### Lack of clinical trial or real-world data

Clinical trials face major challenges, including demonstrating safety and efficacy, managing high costs, recruiting eligible patients, and achieving statistical significance. These challenges are particularly pronounced in nephrology, which has far fewer RCTs compared with cardiology and oncology, despite progressive increase in recent years [21–23]. The systematic exclusion of patients on dialysis from pivotal trials has created a major evidence gap. More than 80% of RCTs exclude ESKD patients, leaving clinicians without evidence-based guidance for commonly treatment across multiple therapeutic areas [23–26].

ESKD patients are excluded at least for two reasons. First, patients treated by dialysis have a reduced life expectancy, which may affect the validity of long-term outcomes. While this concern is relevant for studies exceeding 5 years (e.g. prostate cancer trials in older men), it should not prevent their exclusion from shorter trials of 1–3 years. Second, altered pharmacokinetics, polypharmacy, and a higher comorbidity burden influence clinical trial endpoints. Finally, industry often views patients under dialysis therapy as ‘a niche’ in which the cost of conducting a dedicated RCT is not cost-effective given the potential size of the market.

A substantial proportion of clinical trials conducted in dialysis populations fails to provide sufficient detail regarding the dialysis modality, prescription or adequacy. Reports were often restricted to general statements such as ‘patients were undergoing dialysis’ or at best, ‘patient who has been treated with thrice-weekly haemodialysis for at least three months’ [27, 28]. Yet, dialysis modality and intensity [e.g. standard haemodialysis (HD), HDF, nocturnal or daily dialysis, or peritoneal dialysis] strongly influence drug pharmacokinetics and patient outcomes. Failure to account for these factors constitutes a major methodological limitation.

Finally, only 22% of interventional trials in ESKD populations achieve statistical significance, compared with 59% in cardiovascular trials involving the general population, despite comparable effect sizes [29]. This discrepancy is primarily attributed to inadequate statistical power resulting from smaller sample sizes and fewer conducted RCTs. The limited representativeness of trial population adds to these limitations, as patients on dialysis are typically younger and experience lower mortality rates (8.9 versus 18.6 per 100 patient-years) compared with real-world data (RWD) [29]. Thus, real-world evidence remains limited and fragmented.

Despite recognition for >15 years, this evidence gap persists like the historical exclusion of women from cardiovascular trials. The result is a significant disconnection between clinical trial populations and real-world patients on dialysis, undermining the relevance of research to clinical practice [30].

#### Regulatory guidelines for drug testing in ESKD

During drug development, initial clinical studies typically include only patients with normal or mildly impaired kidney function (eGFR >45 ml/min/1.73 m^2^). In subsequent preregistration studies, only a limited number of patients with more severe kidney dysfunction are included. Data obtained from patients with either severe kidney dysfunction (defined by an eGFR < 30 ml/min/1.73 m^2^, CKD stage 4) or ESKD requiring kidney replacement therapy are particularly limited before drug registration. Only a minority of new drug applications to the US Food and Drug Administration (FDA) include data from this population.

Both agencies FDA and the European Medicines Agency (EMA) have published detailed guidelines for conducting trials in patients with renal impairment, including those on dialysis. A renal impairment study is mandatory and may be required for Marketing Authorization Application. An updated position in the FDA draft guidelines states that patients with severely impaired kidney function may be suitable for reduced study requirements [31]. Under certain circumstances, population pharmacokinetic and pharmacodynamic, modelling analysis may provide sufficient information to inform renal impairment labelling.

### Use of theoretically ‘contraindicated’ drugs in patients on dialysis: lessons from RWD and RCT

Despite formal contraindications, several pharmacological agents have been investigated or used in patients on dialysis, with emerging evidence suggesting potential therapeutic benefits when appropriate adaptations are implemented (see Tables 1 and 2). For each drug, we have defined the level of evidence based on our literature search, that might differ from expert groups.

**Table 1:** Summary of trial/studies that have tested off-label use of drugs in dialysis patients.

Drug	First FDA approval	Metabolism	Theoretically contraindication if eGFR	Main reason	POC in haemodialysis	Date	Reference
**Antiviral drug**							
Sofosbuvir	2013	Primary hepatic but main metabolic excreted by kidney	<30 ml/min	Lack of data from RCTs Risk of cardiac toxicities	12 patients (11 HD, 1 DP) were successfully treated with 200 mg of Sofosbuvir Use of standard dose (=400 mg daily) seems feasible. Meta-analysis in 514 patients confirmed efficacy and safety of Sofosbuvir. https://www.zotero.org/maxtouzot/library	2015 2017 2022	[39] [38] [41]
**Anticoagulant**							
Dabigratran	2010	Renal excretion (80%)	<15–30 ml/min	Risk of accumulation and bleeding	Retrospective study on 281 patients treated with Dabigatran compared with 8064 patients treated with Warfarin	2015	[49]
Apixiban	2012	Renal excretion (25%)	<15–30 ml/min	Risk of accumulation and bleeding	Retrospective study on 2351 patients treated with Apixaban compared with 23 172 patients treated with Warfarin First RCT in HD (Apixaban N = 82, Warfarin N = 72) but failed to reach conclusion due to earlier stop because of enrolment challenges	2018 2022	[42] [47]
Rivaroxaban	2011	Renal excretion (33%)	<15–30 ml/min	Risk of accumulation and bleeding	Retrospective study on 244 patients treated compared with 8064 patients treated with Warfarin First RCT in HD (Rivaroxaban N = 46, Warfarin N = 44) showing same efficacy and safety	2015 2020	[49] [6]
**Anti-thrombotics**							
Enoxaparin	1993	Renal elimination	Dose reduction if eGFR <30 ml/min	Risk of accumulation and bleeding	First study with patients with various renal impairment Dose of 1 mg/kg seems safe	2005	[82]
Tinzaparin	2000	Significant non-renal elimination due to higher molecular weight		Lower risk of accumulation than others LMWH	Retrospective PK study in 623 HD patients receiving prophylactic or therapeutic Tinzaparin Tinzaparin PK was not affected by severe renal impairment	2024	[86]
**Bone metabolism drug**							
Alendronate	1995	Primary renal elimination	<30 ml/min	Risk of adynamic bine	First PK study in 6 HD patients	2019	[51]
Denosumab	2013	Degraded by reticuloendothelial system	No dose adjustment needed for renal impairment	Risk of hypocalcaemia (25%–42%) Increased MACE (up to 36%)	Meta-analysis of 84 patients with CKD Stage 4–5 Largest retrospective study on 1523 females HD patients showing 41% of hypocalcaemia	2018 2024	[56] [53]
Romosozumab	2019	Degraded by reticuloendothelial system	No dose adjustment needed for renal impairment	Risk of vascular calcification worsening Risk of MACE	First PK in HD patients (N = 8) compared with patients with renal impairment (N:8) and patients with normal function (N = 8)	2022	[65]
Teriparatide	2019	Hepatic and peripheral metabolism	No dose adjustment needed for renal impairment	Risk of hypocalcaemia Risk of osteosarcoma	First pilot therapy in HD with adynamic bone proved by biopsy comparing Teripatide (N = 9), cinacalcet (N = 11), and Ibrandonate (N = 11)	2012	[60]

Abbreviations: DP, dialysis peritoneal; ARB, angiotensin receptor blockers; ACE, angiotensin converting enzyme.

**Table 2:** Summary of trial/studies that have tested off-label use of cardiovascular/metabolic drugs in dialysis patients.

Drug	First FDA approval	Metabolism	Theoretically contraindication if eGFR	Main reason	POC in haemodialysis	Date	Reference
**Cardiovascular drug**							
Spironolactone	1960	Hepatic	Use with caution with severe stage 4–5 CKD	Risk of hyperkalaemia and hypotension	Spin-D trail RCT including 129 HD with placebo (*n* = 51) vs spironolactone 12.5 mg (*n* = 27), 25 mg (*n* = 26), or 50 mg (*n* = 25) Hyperkalaemia (>6.5 mmol) was similar between spironolactone and placebo with an increase event with 50 mg dose MiREnDa RCT including 97 HD [placebo *n* = 47 vs spironolactone 50 mg daily (*n* = 50)]. Spironolactone increased the frequency of moderate but not severe hyperkalaemia. ALCHEMSIT RCT, event-driven trial included 823 patients on dialysis with high risk of adverse cardiovascular outcomes. No benefit on cardiovascular (MACE) outcomes	2019 2019 2025	[2] [3] [71]
Sacubitril/valsartan	2015	Hepatic metabolism, metabolites have renal elimination	Dose reduction if eGFR <30 ml/min	Concerns about efficacy and safety (hypotension and hyperkalaemia)	Retrospective propensity score-matched comparative study including 1434 sacubitril-valsartan users vs 1434 ACE/ARB users. Compared with ACE/ARB, sacubitril-valsartan was not associated with increased risk of hypotension nor hyperkalaemia.	2024	[75]
**Anti-diabetic drugs**							
GLP1RA Liraglutide Semaglutide	2010 2017	Non-renal Proteolysis	< 30 ml/min	Lack of data	First pilot study on PK in 10 Japanese T2D obese patients First RCT with 20 HD T2D HD patients reporting safety and efficacy of liraglutide compared with control participants Prospective monocentric study on 13 Obese DP patients treated 12 weeks with semaglutide up to 1.0 mg/week. Observational national cohort study with T2D dialysis patients on HD. 6747 out of 151.649 were treated with GLP1-RA. Reduction of 23% of mortality risk and 66% of higher change for waitlisting	2014 2015 2024 2025	[88] [89] [91] [92]
SGLT2i Dapagliflozin Empaglifozin		Primarily renal elimination	<20 ml/min	Lack of data	First PK study in dialysis patients (HD/DP) showing that dapagliflozin was well tolerated, was slightly dialyzable, with non-accumulating pharmacokinetic properties.	2023	[105]

Abbreviations: DP, dialysis peritoneal; ARB, angiotensin receptor blockers; ACE angiotensin converting enzyme,

#### Antiviral drugs (Level of evidence III)

Sofosbuvir (SOF), a direct-acting antiviral for hepatitis C virus (HCV), is a leading example of a drug initially considered as challenging in dialysis patients but later showed to be highly effective in clinical trials.

SOF, a nucleotide HCV polymerase inhibitor, is included in several all-oral regimens. Its main metabolite, GS-331007, is renally excreted and accumulates 5- to 20-fold in patients with advanced CKD or those on dialysis [32, 33]. Although SOF shows low overall toxicity, preclinical studies demonstrated cardiac toxicity at exposures 29 times higher than clinical levels, raising concerns about its safety in patients with ESKD. However, a phase 2b conducted in 32 patients with severe renal impairment but not on dialysis shows sustained virologic response rates comparable to those with normal kidney function without safety issues [34]. These results were confirmed after in a larger meta-analysis [35]. In HD patients, several prospective studies with small size (*N* = 10 to 62) have reported high sustained virologic response rates at both standard and reduced doses, without additional adverse effects [36–40]. Recent meta-analysis of 20 studies (*N* = 514) confirmed the efficacy and safety profile of such strategy [41]. Despite initial FDA labelling excluding those with low eGFR, off-label SOF use expanded post-approval and can be used in this population.

#### Anticoagulation

##### Direct oral anticoagulants (DOACs) (Level of evidence II)

DOACs were initially contraindicated in patients on dialysis due a theoretical ‘increased bleeding risk’. However, recent studies have challenged this initial assumption. DOACs have since demonstrated efficacy and safety comparable to vitamin K antagonists such as warfarin in preventing thromboembolic stroke and are now widely used, offering advantages such as fixed dosing and no need for therapeutic drug monitoring.

While DOACs have been shown to be non-inferior to warfarin in patients with mild-to-moderate CKD, their renal clearance varies depending on the compounds [dabigatran (80%), rivaroxaban (33%), apixaban (25%)] [42, 43]. Data on their use in patients with stage 5 CKD not yet on dialysis (eGFR <15 ml/min/1.73 m^2^) or in those receiving dialysis remain limited [44]. Because of this, pivotal trials initially excluded ESRD patients [45, 46].

Two RCTs compared the safety and efficacy of rivaroxaban (VALKYRIE study, *N* = 132) or apixaban (Renal-AF study, *N* = 154) versus warfarin [6, 47]. The Renal-AF study stopped prematurely because enrolment challenges and was inadequate powered to draw definite conclusions. However, the VALKYRIE instead show less bleeding compared with warfarin. Other reports including a large retrospective study of the US Database confirmed that both drugs, offer similar stroke protection with a potentially better safety profile than vitamin K antagonists [44, 48–50]. Pharmacokinetic studies show that apixaban levels are 26%–48% lower when taken before dialysis compared with after, informing optimal timing and dosing strategies [4, 5]. Clinical data support adjusted regimens, e.g. apixaban 5 mg twice daily or rivaroxaban 10 mg daily in this population [6, 47].

#### Bone metabolic disease

##### Bisphosphonates (Level of evidence III)

Although bisphosphonates are widely used to treat osteoporosis and fragility fractures in the general population, evidence supporting their use in patients on dialysis is limited. Patients on dialysis therapy are typically excluded from trials evaluating new osteoporosis drugs, mainly for two reasons: renal safety concerns and unpredictable bias introduced by the type of renal osteodystrophy.

Alendronate is partially dialyzable, with ∼51.8% removed during haemodialysis, resulting in an elimination rate, such as that observed in patients with normal renal function [51]. It is generally contraindicated in advanced CKD due to its renal clearance and associated risk adynamic bone disease. However, recent studies including and multicentric prospective controlled study (*N* = 48) have shown that alendronate improves bone mineral density (BMD), reduces circulating biomarkers of bone turnover, and reduces the risk of fractures in HD patients with osteoporosis [51–53]. Interestingly, when compared with denosumab (41%), bisphosphonates are associated with a lower incidence of drug-induced hypocalcaemia (2%) [53]. Other agents such as ibandronate should be used cautiously because of the risk of accumulation [54].

The KDIGO guidelines do not support routine use of alendronate in patients on dialysis due to the risk of adynamic bone disease and the lack of robust evidence. Use should be limited to cases of confirmed high-turnover bone disease, the duration of therapy should be shorter, and only under expert supervision. If used, no dose adjustment is recommended for dialysis, but close monitoring is essential. The intravenous formulations will be preferred in patients without residual renal function and no renal risk, but also to avoid interference with intestinal phosphate binders and to ensure compliance. As in the general population, femoral BMD and FRAX score should be reassessed after 3 years of therapy. In summary, the efficacy and safety of bisphosphonates in ESKD patients require further investigation.

##### Denosumab (Level of evidence III)

Denosumab, a monoclonal antibody against RANKL, represents a unique therapeutic option for patients on dialysis with osteoporosis as unlike bisphosphonates, it does not require renal dose adjustment. Denosumab necessitates vigilant monitoring of serum calcium and parathyroid hormone levels to mitigate the risk of hypocalcaemia ranging from 25% to 52%, and SHPT in the dialysis population [53, 55, 56].

Recent studies in patients on dialysis have shown an increase of up to 5% in lumbar and femoral neck BMD after 1 year of denosumab therapy. The gain in bone mass with denosumab is associated with a 45% reduction in the risk of fragility fractures in patients on dialysis [52].

However, in this Japanese observation study (*N* = 1032), denosumab appears to increase the risk for major adverse cardiovascular events (MACE) by 36% [52].

Another issue with denosumab is the offset of its bone effects. Whereas bisphosphonates are retained by the skeleton and treatment cessation is associated with slow bone release, for denosumab the bone loss is rapid. All the bone gain on therapy at the hip is lost within 6 months and associated with a 30% increase in vertebral fractures in osteoporotic women [57]. For these reasons, it is recommended that denosumab should either be administered continuously or followed by some sequential anabolic or antiresorptive therapy.

### PTH and PTH-related peptide (PTHrP) analogues (teriparatide and abaloparatide) (Level of evidence IV)

These two PTH analogues, by binding and activating the PTH/PTHrP receptor type 1 (PTHR1), stimulate both bone formation and resorption, whereby intermittent administration leads to an increase of bone mass. Abaloparatide, an analogue of PTHrP, was designed to have relatively greater affinity for the PTHR1, thus potentially being more anabolic than teriparatide.

Teriparatide has not been adequately studied in patients with creatinine clearance <30 ml/min or those on dialysis [58, 59]. The risk of osteosarcoma limits their use to a maximal duration of 2 years. Small non-controlled retrospective studies have tested teriparatide in patients on dialysis with low serum PTH or with histologically proven adynamic bone disease [60–62]. Clinical data on abaloparatide are still lacking in patients on dialysis [63].

#### Romosozumab (Level of evidence V)

Romosozumab is a human monoclonal antibody against sclerostin. Sclerostin is produced by osteocytes and negatively regulates bone formation rate through the inhibition of Wnt signalling pathway. Romosozumab is not formally contraindicated in patients receiving dialysis therapy, but its safety and efficacy in patients with severe renal impairment or those on dialysis have not been well established [58, 59]. Nevertheless, pharmacodynamic studies have shown that romosozumab exposure was 31% and 43% higher in ESRD compared with patients with normal renal function [64].

There is a theoretical risk that by mobilizing calcium from bone, romosozumab could exacerbate vascular calcification: a major issue in patients on dialysis. Romosozumab carries also a boxed warning for increased risk of myocardial infarction, stroke, and cardiovascular death in patients who had osteoporosis without renal failure: complications that are markedly increased in patients with ESKD. However, a few case studies have suggested that romosozumab may improve BMD in patients on dialysis with adynamic bone disease or high fracture risk [65].

#### Cardiovascular drugs

##### Spironolactone (level I)

The mineralocorticoid receptor antagonist (MRA) spironolactone has been shown to improve cardiac function and reverse left ventricular hypertrophy in patients with heart failure (HF), but there are no consistent findings regarding its efficacy and safety in patients on dialysis [66–68].

Despite theoretical concerns regarding hyperkalaemia in the context of renal dysfunction, controlled studies have demonstrated comparable potassium homeostasis between spironolactone-treated patients on dialysis and control participants [69].

Two prospectives RCTs, the Spin-D (Safety and Cardiovascular Efficacy of Spironolactone in Dialysis-Dependent End-Stage Renal Disease) and the MiREnDa (Mineralocorticoid Receptor Antagonists in End-Stage Renal Disease) trials has provided the reassuring evidence that spironolactone up to 25 mg/day is reasonably safe when patients are properly monitored [2, 3]. Since then, two trials using such strategies of dosing have evaluating the effect of spironolactone on hard endpoints such as mortality, HF, and cardiac remodelling but failed to meet primary endpoints [70, 71]. The ACHIEVE trial showed that spironolactone at the dose of 25 mg daily did not reduce the composite outcome of cardiovascular death or HF hospitalization, while increasing the risk of hyperkalaemia [70]. ALCHEMIST was a multicentre, double-blind, randomized, placebo-controlled, event-driven trial that included 823 patients on dialysis with high risk of adverse cardiovascular outcomes. The trial failed to show a reduction in the incidence of MACE after a median follow-up of 32.6 months [71].

While MRA use in CKD stage 4–5 is associated with absolute reduction in the risk of cardiovascular events in patients, based on the recent pooled analysis from 2 RCTS, results from ACHIEVE and ALCHEMIST trial do not support the off-label use or spironolactone in patients under dialysis therapy [70–72].

##### Sacubitril/valsartan (Level of evidence III)

Sacubitril-valsartan reduces the risk of mortality and hospitalization in patients with HF with reduced ejection fraction (HFrEF) and is approved since 2015 in HFrEF [73]. Sacubitril-valsartan is generally contraindicated or not recommended in patients with ESRD or severe renal impairment (eGFR <30 ml/min/1.73 m²) due to several concerns, including mainly the risk of hyperkalaemia and hypotension [74]. Thus, sacubitril-valsartan may have therapeutic potential with significant clinical implications for patients with HFrEF requiring dialysis. The FDA label and the clinical guidelines do not recommend variables dose of sacubitril/valsartan in patients on dialysis. Recently, a retrospective comparative effectiveness study using including 1343 patients under sacubitril-valsartan showed the safety and safety of this drug in a subgroup of patients on dialysis with the standard dose (24 to 26 mg) [74–76].

##### Statin (Level of evidence I)

Statins are a cornerstone in the prevention of cardiovascular events in the general population. The 2012 KIDGO recommends lipid lowering therapying in all CKD not on dialysis. Haemodialysis itself does not significantly affect statin clearance. While subtle difference might exist in the metabolic process of specific statins, the levels of statins such as atorvastatin are comparable to those measured in the healthy volunteers [77]. Statins are generally well tolerated in haemodialysis patients with minimal adverse events reported and effectively reduced lipid parameters [78]. However, this does not translate into clinical benefit as two large clinical trials (AURORA and 4D trials) showed no significant reduction in cardiovascular events, despite significant LDL cholesterol lowering [79, 80]. Several factors might explain these counterintuitive results. First, complex lipid abnormalities in patients may respond less effectively to statins. Second, persistent inflammation can activate intracellular cholesterol synthesis activated which is not fully inhibited by statins. The latter lead to vascular wall lipid accumulation despite adequate plasma cholesterol reduction known as ‘statin resistance’ [81].

Consequently, the KDIGO guidelines do not recommend initiating statins in dialysis-dependent patients. Nevertheless, many nephrologists continue statin therapy in patients on dialysis, who were treated before dialysis initiation, reflecting the uncertainty and heterogeneity in real-world practice.

#### Antithrombotic medications (enoxaparin, tinzaparin) (Level of evidence IV)

As low-molecular-weight heparin (LMWH) are primarily eliminated via renal pathways, they are usually contraindicated in patients with severe CKD. However, as they are part of specific procedures to avoid vascular thromboses, they are still administered to 22.3% of patients on dialysis undergoing percutaneous coronary interventions, and this despite formal contraindications [82]. While associated with elevated bleeding risk, modified dosing strategies and enhanced monitoring protocols may mitigate these concerns.

Enoxaparin can still be used on significant dose reduction (1 mg/kg once daily, instead of twice daily) for therapeutic dosing and an appropriate anti-Xa monitoring (target peak levels 0.5–1.0 IU/ml) [83, 84].

Furthermore, more recent LMWH such as tinzaparin that could be used up eGFR <15 min/min represents an alternative for short term anticoagulation. Indeed, because of their higher molecular weight they rely less on renal clearance but much more on endothelial hepatic metabolism and elimination [85]. Tinzaparin represents a good alternative to the widely used enoxaparin, but clinical and pharmacological data are lacking. According to the EMA, full dose tinzaparin can be used in patients with eGFR between 20 and 30 ml/min with monitoring of anti-Xa activity (value <1.50 U/ml). Tinzaparin is not recommended below 20 ml/min [85]. However, a recent monocentric retrospective study has shown that tinzaparin pharmacokinetics profiles were not affected by renal impairment in patient with severe CKD and in patients on dialysis [86]. Further studies are needed to confirm these interesting results.

## FUTURE DRUGS/PERSPECTIVES

### Glucagon-like peptide-1 receptor agonists (Level of evidence III)

The glucagon-like peptide-1 receptor agonists (GLP-1RAs) have dramatically changed the management of type 2 diabetes (T2D). GLP-1RAs not only allows to achieve a sustained glycaemic control with a low risk of hypoglycaemia but also improve cardiometabolic risk factors and induce significant total body weight loss. However, their use is currently not recommended in patients with an eGFR <15 ml/min/1.73 m^2^ due to the lack of sufficient data.

GLP1-RAs can be classified into two groups: incretin mimetics and analogues of human GLP-1. The former are eliminated mainly by the kidneys; therefore, their clearance is reduced in patients with CKD. The latter are degraded via peptidases in various tissues, so their elimination does not depend on renal function, making them appropriate to be used in patients on dialysis [87].

Liraglutide was the first GLP-1RAs studied in dialysis. It is a small peptide, completely degraded by proteolysis and thus not eliminated by the kidney. Few studies have assessed the safety and efficacy of GLP-1RAs such as liraglutide in patients on dialysis with T2D [88, 89]. Compared with participants with normal renal function, these studies reported a 50% increase of plasma level of liraglutide and a similar frequency of side effects for a dose of 1.8 mg. At least three small prospectives studies including one with a control group, have confirmed the efficacy and safety of liraglutide in patients on dialysis compared with participants who did not have CKD [88, 90]. Similar results were recently observed with semaglutide [91]. Recently, a retrospective US cohort study involving 6474 out of 808 000 diabetic patients (0.8%) showed that GLP-1RAs were associated with a 23% reduction in mortality and a 66% higher chance of being transplantation waitlisted [92]. It should be noted, however, that one out of three patients experienced worsening diabetic retinopathy. No other significant adverse events were reported. While early data are promising, routine use in dialysis patients should remain cautious until more robust evidence from RCT is available.

### SGLT2 inhibitors (Level of evidence V)

SGLT2 inhibitors (SGLT2i) have recently emerged as uniquely beneficial drugs, demonstrating strong tolerability and significant prognostic benefits for both cardiovascular and renal outcomes in patients with HF [93–97]. These effects have been consistent across all baseline levels of eGFR, including those below 30 ml/min/1.73 m² [98–101]. Although not currently recommended for patients with an eGFR below 20 ml/min/1.73 m², recent observational data suggest that SGLT2i use in this population—particularly in individuals with T2D—is associated with a reduced risk of cardiovascular events and CKD progression to dialysis [99, 102–104]. One recent report has shown that in patients on either haemodialysis or peritoneal dialysis that dapagliflozin demonstrated non-accumulating pharmacokinetic properties with minimal dializability (<1% of the administrated dose) with few side effects as in the general population [105].

At present, in the absence of RCT data in patients on dialysis; pending results from ongoing trials such as the DARE-ESKD-2 Trial (NCT05179668); evidence for SGLT2i use in this setting comes from observational sources [106]. For example, patients in the DAPA-HF trial who initiated dialysis during follow up and continued SGLT2i therapy still experienced clinical benefits [93]. Importantly, SGLT2i have also demonstrated favourable effects on reverse cardiac remodelling.

## PERSPECTIVES: OVERCOMING THEORETICAL CONTRAINDICATIONS AND EXTENDING INDICATIONS

**Figure 2:**
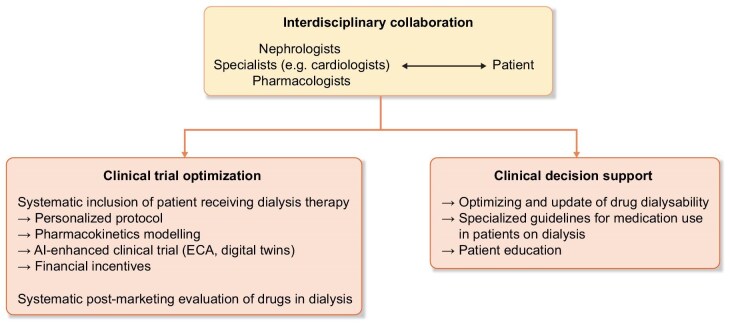
Perspectives to facilitate off-label use and extending indications. Abbreviation: ECA, external control arm.

Categorical contraindications should always be challenged and viewed not as obstacles, but as opportunities to extend drug’s indication. Several aspects to be considered are discussed next and summarized in Fig. 2.

### Precision pharmacokinetic approaches

The implementation of therapeutic drug monitoring, coupled with advanced pharmacokinetic modelling specific to patients on dialysis, represents a promising strategy to individualize dosing regimens [107]. These approaches can account for residual renal function, dialysis characteristics, and patient-specific factors to optimize therapeutic outcomes while minimizing adverse effects. Even in the absence of pharmacokinetic data for certain drugs, a four-step approach proposed by Gotta and Coll might facilitate individualized dose recommendations in patients receiving dialysis therapy [108]. However, this strategy may apply only to a limited number of drugs for which serum concentration monitoring is available. A similar approach could be envisioned for patients with advanced CKD or ESKD.

### Innovations in dialysis technology

Advances in dialysis membrane technology have significantly improved toxin clearance. Drug dialyzability, therefore, should not be considered fixed as it is influenced by the specific dialysis modality employed (e.g. haemodialysis, peritoneal dialysis, or hemodiafiltration) and their duration. For example, expanded haemodialysis (HDx) utilizing medium cut-off membranes offers enhanced clearance of middle molecular weight uremic toxins, potentially modifying the pharmacokinetic profile of certain medications [109]. Personalized dialysis prescriptions, such as daily home haemodialysis and long nocturnal haemodialysis, may further optimize the therapeutic window for traditionally contraindicated agents in this population [110].

### Advanced clinical decision support

As previously mentioned, reference data regarding drug use in patients on dialysis remain inconsistent [18, 111]. Developing specialized guidelines for medication use in patients on dialysis would further standardize approaches to medication management in this population. Several platforms have been developed, most of them focusing primarily on antibiotic dosing. However, only a few, such as SiteGRP.com, have attempted to cover multiple drug classes thereby addressing this gap. This platform provides clinicians with unvaluable tools and resources on nephrotoxicity and drug dosing adjustments in CKD.

### AI-enhanced clinical decision making

AI-based systems can integrate complex, multidimensional data, including patient demographics, comorbidities, laboratory values, dialysis parameters, and treatment histories, to generate personalized dosing recommendations that traditional pharmacokinetic models cannot achieve alone. Machine learning algorithms have demonstrated promising results in predicting drug concentrations during haemodialysis sessions with greater accuracy than conventional methods [112, 113]. Moreover, AI systems can identify patterns across patient populations to detect previously unrecognized drug interactions specific to patients on dialysis and to predict which contraindicated medications might benefit specific subgroups [114]. The integration of AI with electronic health records creates opportunities for real-time clinical decision support, enabling clinicians to receive alerts about potential medication issues before they occur and offering evidence-based alternatives. However, despite its promise, implementation remains challenging due to multiple barriers (model transparency, validation across diverse patient populations, ethical considerations, data privacy...) [114, 115].

### Changes in clinical trial design

Systematic inclusion of patients receiving dialysis therapy in clinical trials is essential to generate robust evidence-based recommendations [116]. Concerns that their inclusion could affect efficacy or safety outcomes can be addressed through thoughtful approaches to studying designs and appropriate risk mitigation strategies. For example, the use of an External control Arm may reduce the number of patients on dialysis required while still maintaining robust signal for treatment efficacy [117, 118]. Digital twins of dialysis patients could improve their representation in pre-marketing authorization trials and support safer, more personalized treatment. Finally, regulatory and financial incentives could serve to mitigate financial concerns with involving patients with kidney disease in cardiovascular trials.

### Interdisciplinary collaboration

Optimal medication management in patients on dialysis requires more than nephrologist oversight, it necessitates collaboration among nephrologists, clinical pharmacists, and other relevant specialists. The decision to prescribe an off-label drug should be guided by a thorough evaluation of the benefits and risks, with all specialists involved. Physicians should align on a well-defined therapeutical strategy to minimize adverse events (dose titration, pharmacodynamic if available, assessment of efficacy with selected endpoints). Depending on the strength of evidence in the literature, clinicians should also consider conducting a clinical trial or at least a prospective study within their own institution. Finally, involvement of the patient early in the treatment process is mandatory! Patient should be informed from the beginning of decision making regarding risk–benefit assessments. Patients need understand the rationale for prescribing the drug (benefit), the theoretical reasons for its contraindication (risk), and how we manage to prescribe it safely (off-label use protocol). Patient education by physician, nurses, and pharmacist is critical, not only to promote adherence but also to encourage reporting any adverse events. Patient reported outcomes should be systematically collected even in the absence of a clinical trial.

**Figure 3:**
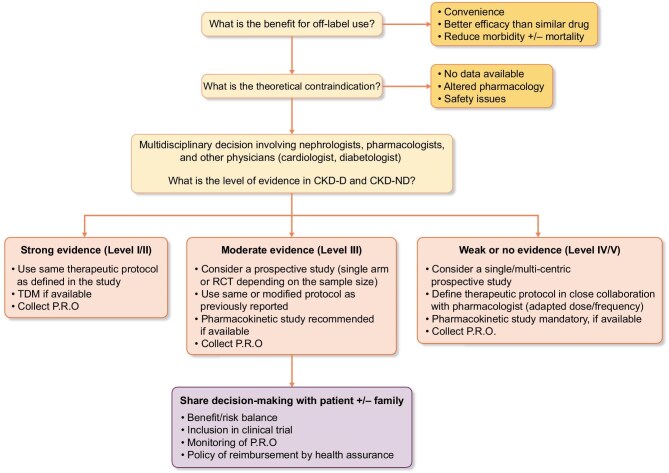
Recommended strategy to be considered prior to the initiation of an off-label drug. Abbreviations: CKD-ND, CKD non-dialysis; CKD-D, CKD on dialysis; PRO, patient reported outcomes; RCT, randomized clinical trial; TDM, therapeutic drug monitoring.

Figure 3 summarizes recommendations before considering the off-label use of drug in this population.

### Policy of reimbursement

Although harmonized marketing authorization requirements exists, there is still a lack of unified policy for off-label drug reimbursement across country or regions in the world. The decision to authorize an off-label use is both country- and drug-specific. Several countries have developed specific reimbursement list of drugs, mainly in oncology.

In the USA, frameworks for off-label cancer drug rely on reference lists, yet nearly half of them remain ultimately non-reimbursed. In France, The RTU (Recommandation Temporaire d’Utilisation) framework allows reimbursable off-label use when ANSM issues an RTU. Physicians in Germany can apply for cost coverage, on a case-by-case basis, prior to treatment initiation.

Similarly, in Italy, the Law 648/1996 created a national AIFA ‘648 list’ including a specific off-label (and other early-access) uses that are reimbursed by the National Health Service when criteria are met.

The main perspectives are summarized in Fig. 2.

## CONCLUSION

The traditional paradigm of categorically contraindicating medications in patients receiving dialysis therapy should be reconsidered in light of emerging clinical and real-world evidence. Pharmacokinetic and pharmacodynamic alterations should not automatically preclude necessary therapeutic interventions but rather inform individualized treatment strategies based on the available evidence. Through the implementation of precision dosing approaches, enhanced monitoring protocols, and interdisciplinary collaboration including the patient, the therapeutic armamentarium for patients on dialysis can be judiciously expanded. Future research initiatives should prioritize generating high-quality evidence to guide safe and effective medication use in this vulnerable population, with particular focus on addressing the significant disparity in clinical trial design compared with other therapeutic areas. Trials such as VALKYRIE and ALCHEMIST show us the way forwards.

## Data Availability

No new data were generated or analysed in support of this research.

## References

[1] Liu J, Jia WY, Yu C. Safety and efficacy of spironolactone in dialysis-dependent patients: meta-analysis of randomized controlled trials. Front Med 2022;9. 10.3389/fmed.2022.828189 PMC8970057 35372414

[2] Charytan DM, Himmelfarb J, Ikizler TA et al. Safety and cardiovascular efficacy of spironolactone in dialysis-dependent ESRD (SPin-D): a randomized, placebo-controlled, multiple dosage trial. Kidney Int 2019;95:973–82. 30473139 10.1016/j.kint.2018.08.034 PMC6431563

[3] Hammer F, Krane V, Störk S et al. Rationale and design of the mineralocorticoid receptor antagonists in end-stage renal disease study (MiREnDa). Nephrol Dial Transplant 2014;29:400–5. 10.1093/ndt/gft409 24166468

[4] Van den Bosch I, Bouillon T, Verhamme P et al. Apixaban in patients on haemodialysis: a single-dose pharmacokinetics study. Nephrol Dial Transplant 2021;36:884–9. 10.1093/ndt/gfaa351 33351142

[5] Konecki C, Lipman ML, Mavrakanas TA et al. Population pharmacokinetic modelling of Apixaban in end-stage kidney disease patients with atrial fibrillation receiving haemodialysis. Clin Pharmacokinet 2025;64:307–21. 10.1007/s40262-025-01476-6 39853633

[6] de Vriese AS, Caluwé R, Pyfferoen L et al. Multicenter randomized controlled trial of Vitamin K antagonist replacement by rivaroxaban with or without vitamin K2 in hemodialysis patients with atrial fibrillation: the Valkyrie study. J Am Soc Nephrol 2020;31:186–96. 10.1681/ASN.2019060579 31704740 PMC6935010

[7] Sun H, Frassetto L, Benet LZ. Effects of renal failure on drug transport and metabolism. Pharmacol Ther 2006;109:1–11. 10.1016/j.pharmthera.2005.05.010 16085315

[8] Velenosi TJ, Urquhart BL. Pharmacokinetic considerations in chronic kidney disease and patients requiring dialysis. Expert Opin Drug Metabol Toxicol 2014;10:1131–43. 10.1517/17425255.2014.931371 24961255

[9] Naud J, Nolin TD, Leblond FA et al. Current understanding of drug disposition in kidney disease. J Clin Pharmacol 2012;52:10S–22S. 10.1177/0091270011413588 22232747

[10] Atkinson AJ, Umans JG. Pharmacokinetic studies in hemodialysis patients. Clin Pharmacol Ther 2009;86:548–52. 10.1038/clpt.2009.147 19675540

[11] Talbert RL. Drug dosing in renal insufficiency. J Clin Pharmacol 1994;34:99–110. 10.1002/j.1552-4604.1994.tb03973.x 8163720

[12] N.J , L.-V. V, S.K et al. Vaccination and chronic kidney disease. Nephrol Dial Transplant 2008;23:800–7. 18065804 10.1093/ndt/gfm851

[13] Lea-Henry TN, Carland JE, Stocker SL et al. Clinical pharmacokinetics in kidney disease: fundamental principles. Clin J Am Soc Nephrol 2018;13:1085–95. 10.2215/CJN.00340118 29934432 PMC6032582

[14] Oosting IJ, Colombijn JMT, Kaasenbrood L et al. Polypharmacy in patients with CKD: a systematic review and meta-analysis. Kidney360 2024;5:841–50. 10.34067/KID.0000000000000447 38661553 PMC11219116

[15] Papotti B, Marchi C, Adorni MP et al. Drug-drug interactions in polypharmacy patients: the impact of renal impairment. Curr Res Pharmacol Drug Discov 2021;2:100020. 10.1016/j.crphar.2021.100020 34909655 PMC8663981

[16] Van Oosten MJM, Logtenberg SJJ, Hemmelder MH et al. Polypharmacy and medication use in patients with chronic kidney disease with and without kidney replacement therapy compared to matched controls. Clin Kidney J 2021;14:2497–523. 10.1093/ckj/sfab120 34950462 PMC8690067

[17] Gagne JJ, Khan NF, Raj TS et al. Strength of evidence for labeled dosing recommendations in renal impairment. Clinical Trials 2017;14:219–21. 10.1177/1740774516673818 27780884

[18] Lewis SJ, Bodenhorn D, Na EY et al. Comparison of antimicrobial dosing recommendations in patients receiving intermittent hemodialysis among drug information resources. J Clin Pharm Ther 2022;47:628–35. 10.1111/jcpt.13583 34866202

[19] Tieu A, Velenosi TJ, Kucey AS et al. β-blocker dialyzability in maintenance hemodialysis patients a randomized clinical trial. Clin J Am Soc Nephrol 2018;13:604–11. 10.2215/CJN.07470717 29519953 PMC5969458

[20] Polkinghorne KR, McMahon LP, Becker GJ. Pharmacokinetic studies of dalteparin (Fragmin), enoxaparin (clexane), and danaparoid sodium (orgaran) in stable chronic hemodialysis patients. Am J Kidney Dis 2002;40:990–5. 10.1053/ajkd.2002.36331 12407644

[21] Strippoli GFM, Craig JC, Schena FP. The number, quality, and coverage of randomized controlled trials in nephrology. J Am Soc Nephrol 2004;15:411–9. 10.1097/01.ASN.0000100125.21491.46 14747388

[22] Chatzimanouil MKT, Wilkens L, Anders HJ. Quantity and reporting quality of kidney research. J Am Soc Nephrol 2019;30:13–22. 10.1681/ASN.2018050515 30545982 PMC6317598

[23] Zoccali C, Blankestijn PJ, Bruchfeld A et al. Children of a lesser god: exclusion of chronic kidney disease patients from clinical trials. Nephrol Dial Transplant 2019;34:1112–4. 10.1093/ndt/gfz023 30815678

[24] Delaye M, Rousseau A, Try M et al. Inclusion of patients with chronic kidney disease in randomized phase 3 clinical trials in patients with prostate, breast, lung, and colorectal cancer. Cancer Med 2023;12:3172–5. 10.1002/cam4.5171 36156460 PMC9939176

[25] Klil-Drori AJ, Tagalakis V. Direct oral anticoagulants in end-stage renal disease. Semin Thromb Hemost 2018;44:353–63. 29320795 10.1055/s-0037-1621715

[26] Sprangers B, Perazella MA, Lichtman SM et al. Improving cancer care for patients with CKD: the need for changes in clinical trials. Kidney Int Rep 2022;7:1939–50. 10.1016/j.ekir.2022.06.005 36090489 PMC9458993

[27] Besarab A, Bolton WK, Browne JK et al. The effects of normal as compared with low hematocrit values in patients with cardiac disease who are receiving hemodialysis and epoetin. N Engl J Med 1998;339:584–90. 10.1056/NEJM199808273390903 9718377

[28] Block GA, Martin KJ, de Francisco ALM et al. Cinacalcet for secondary hyperparathyroidism in patients receiving hemodialysis. N Engl J Med 2004;350:1516–25. 10.1056/NEJMoa031633 15071126

[29] Raggi P, Boer R, Goodman WG et al. Cardiovascular and renal outcomes trials—is there a difference? Am J Cardiol 2015;116:982–8. 10.1016/j.amjcard.2015.06.024 26198118

[30] Garg AX, Iansavichus AV, Kastner M et al. Lost in publication: half of all renal practice evidence is published in non-renal journals. Kidney Int 2006;70:1995–2005. 10.1038/sj.ki.5001896 17035946

[31] Pharmacokinetics in Patients with Impaired Renal Function—Study Design, Data Analysis, and Impact on Dosing | FDA Available from: https://www.fda.gov/regulatory-information/search-fda-guidance-documents/pharmacokinetics-patients-impaired-renal-function-study-design-data-analysis-and-impact-dosing

[32] Sise ME, McQuaid T, Martin P. Sofosbuvir-based hepatitis C therapies in patients with chronic and end-stage kidney disease. Nephrol Dial Transplant 2022;37:2327–34. 10.1093/ndt/gfab072 33848334

[33] Kirby BJ, Symonds WT, Kearney BP et al. Pharmacokinetic, pharmacodynamic, and drug-interaction profile of the hepatitis C virus NS5B polymerase inhibitor sofosbuvir. Clin Pharmacokinet 2015;54:677–90. 10.1007/s40262-015-0261-7 25822283

[34] Lawitz E, Landis CS, Flamm SL et al. Sofosbuvir plus ribavirin and sofosbuvir plus ledipasvir in patients with genotype 1 or 3 hepatitis C virus and severe renal impairment: a multicentre, phase 2b, non-randomised, open-label study. Lancet Gastroenterol Hepatol 2020;5:918–26. 10.1016/S2468-1253(19)30417-0 32531259

[35] Li M, Chen J, Fang Z et al. Sofosbuvir-based regimen is safe and effective for hepatitis C infected patients with stage 4-5 chronic kidney disease: a systematic review and meta-analysis. Virol J 2019;16:34. 10.1186/s12985-019-1140-x 30871566 PMC6419462

[36] Borgia SM, Dearden J, Yoshida EM et al. Sofosbuvir/velpatasvir for 12 weeks in hepatitis C virus-infected patients with end-stage renal disease undergoing dialysis. J Hepatol 2019;71:660–5. 10.1016/j.jhep.2019.05.028 31195062

[37] Mehta R, Desai K, Kabrawala M et al. Preliminary experience with sofosbuvir-based treatment regimens for patients dependent on hemodialysis. Indian J Gastroenterol 2018;37:72–73. 10.1007/s12664-018-0833-1 29430587

[38] He YL, Yang SJ, Hu CH et al. Safety and efficacy of sofosbuvir-based treatment of acute hepatitis C in end-stage renal disease patients undergoing haemodialysis. Aliment Pharmacol Ther 2018;47:526–32. 10.1111/apt.14429 29250808

[39] Kalyan Ram B, Frank C, Adam P et al. Safety, efficacy and tolerability of half-dose sofosbuvir plus simeprevir in treatment of hepatitis C in patients with end stage renal disease. J Hepatol 2015;63:763–5. 10.1016/j.jhep.2015.06.004 26095179

[40] Desnoyer A, Pospai D, Lê MP et al. Pharmacokinetics, safety and efficacy of a full dose sofosbuvir-based regimen given daily in hemodialysis patients with chronic hepatitis C. J Hepatol 2016;65:40–47. 10.1016/j.jhep.2016.02.044 26952005

[41] Shehadeh F, Kalligeros M, Byrd K et al. Efficacy and safety of sofosbuvir in the treatment of hep C among patients on hemodialysis: a systematic review and meta-analysis. Sci Rep 2020;10:14332. 10.1038/s41598-020-71205-5 32868869 PMC7459301

[42] Laugesen EK, Staerk L, Carlson N et al. Non-vitamin K antagonist oral anticoagulants vs. vitamin-K antagonists in patients with atrial fibrillation and chronic kidney disease: a nationwide cohort study. Thromb J 2019;17:21. 10.1186/s12959-019-0211-y 31736658 PMC6849210

[43] Bauer KA. Pros and cons of new oral anticoagulants. Hematology Am Soc Hematol Educ Program 2013;2013:464–70. 10.1182/asheducation-2013.1.464 24319220

[44] Chan KE, Giugliano RP, Patel MR et al. Nonvitamin K anticoagulant agents in patients with advanced chronic kidney disease or on dialysis with AF. J Am Coll Cardiol 2016;67:2888–99. 10.1016/j.jacc.2016.02.082 27311528

[45] Dager WE, Lee JA. Filtering out use of DOACs in hemodialysis. Ann Pharmacother 2017;51:511–3. 10.1177/1060028016689265 28114796

[46] Elfar S, Elzeiny SM, Ismail H et al. Direct oral anticoagulants vs. warfarin in hemodialysis patients with atrial fibrillation: a systematic review and meta-analysis. Front Cardiovasc Med 2022;9:9. 10.3389/fcvm.2022.847286 PMC9218480 35757350

[47] Pokorney SD, Chertow GM, Al-Khalidi HR et al. Apixaban for patients with atrial fibrillation on hemodialysis: a multicenter randomized controlled trial. Circulation 2022;146:1735–45. 10.1161/CIRCULATIONAHA.121.054990 36335914

[48] Siontis KC, Zhang X, Eckard A et al. Outcomes associated with apixaban use in patients with end-stage kidney disease and atrial fibrillation in the United States. Circulation 2018;138:1519–29. 10.1161/CIRCULATIONAHA.118.035418 29954737 PMC6202193

[49] Chan KE, Edelman ER, Wenger JB et al. Dabigatran and rivaroxaban use in atrial fibrillation patients on hemodialysis. Circulation 2015;131:972–9. 10.1161/CIRCULATIONAHA.114.014113 25595139 PMC4363265

[50] Sarratt SC, Nesbit R, Moye R. Safety outcomes of Apixaban compared with Warfarin in patients with end-stage renal disease. Ann Pharmacother 2017;51:445–50. 10.1177/1060028017694654 28478715

[51] Iseri K, Watanabe M, Lee XP et al. Elimination of intravenous alendronate by hemodialysis: a kinetic study. Hemodial Int 2019;23:466–71. 10.1111/hdi.12773 31328884

[52] Masuda S, Fukasawa T, Matsuda S et al. Cardiovascular safety and fracture prevention effectiveness of denosumab versus oral bisphosphonates in patients receiving dialysis : a target trial emulation. Ann Intern Med 2025;178:167–76. 10.7326/ANNALS-24-03237 39761590

[53] Bird ST, Smith ER, Gelperin K et al. Severe hypocalcemia with denosumab among older female dialysis-dependent patients. JAMA 2024;331:491–9. 10.1001/jama.2023.28239 38241060 PMC10799290

[54] Bergner R. Bisphosphonate therapy in renal osteodystrophy—a review. J Nephrol 2013;26:450–5. 10.5301/jn.5000188 22760876

[55] Jang SM, Anam S, Pringle T et al. Contrasting PTH response of denosumab use in dialysis patients: a report of 2 cases. pharm 2020;8:59. 10.3390/pharmacy8020059 PMC7355881 32244607

[56] Thongprayoon C, Acharya P, Acharya C et al. Hypocalcemia and bone mineral density changes following denosumab treatment in end-stage renal disease patients: a meta-analysis of observational studies. Osteoporos Int 2018;29:1737–45. 10.1007/s00198-018-4533-6 29713798

[57] Cummings SR, Ferrari S, Eastell R et al. Vertebral fractures after discontinuation of denosumab: a post hoc analysis of the randomized placebo-controlled FREEDOM trial and its extension. J Bone Miner Res 2018;33:190–8. 29105841 10.1002/jbmr.3337

[58] Brown JP, Engelke K, Keaveny TM et al. Romosozumab improves lumbar spine bone mass and bone strength parameters relative to alendronate in postmenopausal women: results from the Active-Controlled Fracture Study in Postmenopausal Women with Osteoporosis at High Risk (ARCH) Trial. J Bone Miner Res 2021;36:2139–52. 34190361 10.1002/jbmr.4409 PMC9292813

[59] Cosman F, Crittenden DB, Ferrari S et al. FRAME study: the foundation effect of building bone with 1 year of romosozumab leads to continued lower fracture risk after transition to denosumab. J Bone Miner Res 2018;33:1219–26. 10.1002/jbmr.3427 29573473

[60] Mitsopoulos E, Ginikopoulou E, Economidou D et al. Impact of long-term cinacalcet, ibandronate or teriparatide therapy on bone mineral density of hemodialysis patients: a pilot study. Am J Nephrol 2012;36:238–44. 10.1159/000341864 22948280

[61] Cejka D, Kodras K, Bader T et al. Treatment of hemodialysis-associated adynamic bone disease with teriparatide (PTH1-34): a pilot study. Kidney Blood Pressure Res 2010;33:221–6. 10.1159/000316708 20588059

[62] Yamamoto J, Nakazawa D, Nishio S et al. Impact of weekly teriparatide on the bone and mineral metabolism in hemodialysis patients with relatively low serum parathyroid hormone: a pilot study. Therapeutic Apheresis Dialysis 2020;24:146–53. 10.1111/1744-9987.12867 31210004

[63] Bilezikian JP, Hattersley G, Mitlak BH et al. Abaloparatide in patients with mild or moderate renal impairment: results from the ACTIVE phase 3 trial. Curr Med Res Opin 2019;35:2097–102. 10.1080/03007995.2019.1656955 31418585

[64] Hsu CP, Maddox J, Block G et al. Influence of renal function on pharmacokinetics, pharmacodynamics, and safety of a single dose of Romosozumab. J Clin Pharmacol 2022;62:1132–41. 35304747 10.1002/jcph.2050 PMC9542825

[65] Suzuki T, Mizobuchi M, Yoshida S et al. Romosozumab successfully regulated progressive osteoporosis in a patient with autosomal dominant polycystic kidney disease undergoing hemodialysis. Osteoporos Int 2022;33:2649–52. 35980440 10.1007/s00198-022-06534-4

[66] Edwards NC, Steeds RP, Stewart PM et al. Effect of spironolactone on left ventricular mass and aortic stiffness in early-stage chronic kidney disease. A randomized controlled trial. J Am Coll Cardiol 2009;54:505–12. 19643310 10.1016/j.jacc.2009.03.066

[67] Mottram PM, Haluska B, Leano R et al. Effect of aldosterone antagonism on myocardial dysfunction in hypertensive patients with diastolic heart failure. Circulation 2004;110:558–65. 15277317 10.1161/01.CIR.0000138680.89536.A9

[68] Udelson JE, Feldman AM, Greenberg B et al. Randomized, double-blind, multicenter, placebo-controlled study evaluating the effect of aldosterone antagonism with eplerenone on ventricular remodeling in patients with mild-to-moderate heart failure and left ventricular systolic dysfunction. Circ Heart Fail 2010;3:347–53. 20299607 10.1161/CIRCHEARTFAILURE.109.906909

[69] Chua D, Lo A, Lo C. Spironolactone use in heart failure patients with end-stage renal disease on hemodialysis: is it safe? Clin Cardiol 2010;33:604–8. 10.1002/clc.20838 20960534 PMC6653125

[70] Walsh M, Collister D, Gallagher M et al. The Aldosterone blockade for health improvement evaluation in end-stage renal disease (ACHIEVE) trial: rationale and clinical research protocol. Can J Kidney Health Dis 2025;3:12. 10.1177/20543581251348187 PMC12134517 40470466

[71] Rossignol P, Zannad F, Massy Z et al. Spironolactone in patients on chronic haemodialysis at high risk of adverse cardiovascular outcomes (ALCHEMIST): a multicentre, double-blind, randomised, placebo-controlled trial and updated meta-analysis. Lancet 2025;406:705–18. 10.1016/S0140-6736(25)01194-8 40818851

[72] Matsumoto S, Henderson AD, Shen L et al. Mineralocorticoid receptor antagonists in patients with heart failure and impaired renal function. J Am Coll Cardiol 2024;83:2426–36. 10.1016/j.jacc.2024.03.426 38739064

[73] Solomon SD, Vaduganathan M, Claggett BL et al. Sacubitril/valsartan across the spectrum of ejection fraction in heart failure. Circulation 2020;141:352–61. 10.1161/CIRCULATIONAHA.119.044586 31736342

[74] Chatur S, Beldhuis IE, Claggett BL et al. Sacubitril/valsartan in patients with heart failure and deterioration in eGFR to <30 mL/min/1.73 m^2^. JACC: Heart Fail 2024;12:1692–1703. 38842957 10.1016/j.jchf.2024.03.014

[75] Le D, Grams ME, Coresh J et al. Sacubitril-valsartan in patients requiring hemodialysis. JAMA Netw Open 2024;7:e2429237. 10.1001/jamanetworkopen.2024.29237 39163041 PMC11337068

[76] Kang H, Zhang J, Zhang X et al. Effects of sacubitril/valsartan in patients with heart failure and chronic kidney disease: a meta-analysis. Eur J Pharmacol 2020;884:173444. 10.1016/j.ejphar.2020.173444 32739172

[77] Lins RL, Matthys KE, Verpooten GA et al. Pharmacokinetics of atorvastatin and its metabolites after single and multiple dosing in hypercholesterolaemic haemodialysis patients. Nephrol Dial Transplant 2003;18:967–76. 10.1093/ndt/gfg048 12686673

[78] Nishikawa O, Mune M, Miyano M et al. Effect of simvastatin on the lipid profile of hemodialysis patients. Kidney Int Suppl 1999;56:S219–21. 10.1046/j.1523-1755.1999.07157.x 10412781

[79] Fellström BC, Jardine AG, Schmieder RE et al. Rosuvastatin and cardiovascular events in patients undergoing hemodialysis. N Engl J Med 2009;360:1395–407. 10.1056/NEJMoa0810177 19332456

[80] Wanner C, Krane V, März W et al. Atorvastatin in patients with type 2 diabetes mellitus undergoing hemodialysis. N Engl J Med 2005;353:238–48. 10.1056/NEJMoa043545 16034009

[81] Chen Y, Ku H, Zhao L et al. Inflammatory stress induces statin resistance by disrupting 3-hydroxy-3-methylglutaryl-CoA reductase feedback regulation. Arterioscler Thromb Vasc Biol 2014;34:365–76. 10.1161/ATVBAHA.113.301301 24233489

[82] Tsai TT, Maddox TM, Roe MT et al. Contraindicated medication use in dialysis patients undergoing percutaneous coronary intervention. JAMA 2009;302:2458–64. 10.1001/jama.2009.1800 19996401

[83] Green B, Greenwood M, Saltissi D et al. Dosing strategy for enoxaparin in patients with renal impairment presenting with acute coronary syndromes. Br J Clin Pharmacol 2005;59:281–90. 10.1111/j.1365-2125.2004.02253.x 15752373 PMC1884796

[84] Lachish T, Rudensky B, Slotki I et al. Enoxaparin dosage adjustment in patients with severe renal failure: antifactor xa concentrations and safety. Pharmacotherapy 2007;27:1347–52. 10.1592/phco.27.10.1347 17896889

[85] Inhixa | European Medicines Agency (EMA). https://www.ema.europa.eu/en/medicines/human/EPAR/inhixa (date last accessed 3 June 2025).

[86] Gouin-Thibault I, Mansour A, Caribotti C et al. Tinzaparin, an alternative to subcutaneous unfractionated heparin, in patients with severe and end-stage renal impairment: a retrospective observational single-center study. J Thromb Haemost 2024;22:2864–72. 10.1016/j.jtha.2024.07.006 39019439

[87] Marbury TC, Flint A, Jacobsen JB et al. Pharmacokinetics and tolerability of a single dose of semaglutide, a Human glucagon-like peptide-1 analog, in subjects with and without renal impairment. Clin Pharmacokinet 2017;56:1381–90. 10.1007/s40262-017-0528-2 28349386 PMC5648736

[88] Osonoi T, Saito M, Tamasawa A et al. Effect of hemodialysis on plasma glucose profile and plasma level of liraglutide in patients with type 2 diabetes mellitus and end-stage renal disease: a pilot study. PLoS ONE 2014;9:e113468. 10.1371/journal.pone.0113468 25526642 PMC4272272

[89] Idorn T, Knop FK, Jørgensen MB et al. Safety and efficacy of liraglutide in patients with type 2 diabetes and end-stage renal disease: an investigator-initiated, placebo-controlled, double-blinded, parallel group, randomized trial. Diabetes Care 2015;39:206–13. 26283739 10.2337/dc15-1025

[90] Touzot M, Voican A, Potier L et al. Efficacy and tolerance of liraglutide for weight loss in obese, type 2 diabetes and haemodialysis patients. Diabetes Obes Metab 2025;27:4599–4602. 40432380 10.1111/dom.16484

[91] Vanek L, Kurnikowski A, Krenn S et al. Semaglutide in patients with kidney failure and obesity undergoing dialysis and wishing to be transplanted: a prospective, observational, open-label study. Diabetes Obes Metab 2024;26:5931–41. 39375862 10.1111/dom.15967

[92] Orandi BJ, Chen Y, Li Y et al. GLP-1 receptor agonist outcomes, safety, and BMI change in a national cohort of dialysis patients. Clin J Am Soc Nephrol 2025;20:1100–10. 10.2215/CJN.0000000750 40526425 PMC12342107

[93] Solomon SD, McMurray JJV, Claggett B et al. Dapagliflozin in heart failure with mildly reduced or preserved ejection fraction. N Engl J Med 2022;387:1089–98. 10.1056/NEJMoa2206286 36027570

[94] Ji Pj, Zhang Zy, Yan Q et al. The cardiovascular effects of SGLT2 inhibitors, RAS inhibitors, and ARN inhibitors in heart failure. ESC Heart Fail 2023;10:1314–25. 36722326 10.1002/ehf2.14298 PMC10053170

[95] McMurray JJV, Wheeler DC, Stefánsson BV. et al. Effects of dapagliflozin in patients with kidney disease, with and without heart failure. JACC: Heart Fail 2021;9:807–20. 34446370 10.1016/j.jchf.2021.06.017

[96] Solomon SD, de Boer RA, DeMets D et al. Dapagliflozin in heart failure with preserved and mildly reduced ejection fraction: rationale and design of the DELIVER trial. Eur J Heart Fail 2021;23:1217–25. 34051124 10.1002/ejhf.2249 PMC8361994

[97] McMurray JJV, DeMets DL, Inzucchi SE et al. A trial to evaluate the effect of the sodium–glucose co-transporter 2 inhibitor dapagliflozin on morbidity and mortality in patients with heart failure and reduced left ventricular ejection fraction (DAPA-HF). Eur J Heart Fail 2019;21:665–75. 30895697 10.1002/ejhf.1432 PMC6607736

[98] Wheeler DC, Stefánsson BV, Jongs N et al. Effects of dapagliflozin on major adverse kidney and cardiovascular events in patients with diabetic and non-diabetic chronic kidney disease: a prespecified analysis from the DAPA-CKD trial. Lancet Diabetes Endocrinoly 2021;9:22–31. 10.1016/S2213-8587(20)30369-7 33338413

[99] Heerspink HJL, Jongs N, Chertow GM et al. Effect of dapagliflozin on the rate of decline in kidney function in patients with chronic kidney disease with and without type 2 diabetes: a prespecified analysis from the DAPA-CKD trial. Lancet Diabetes Endocrinol 2021;9:743–54. 10.1016/S2213-8587(21)00242-4 34619108

[100] Jongs N, Chertow GM, Greene T et al. Correlates and consequences of an acute change in eGFR in response to the SGLT2 inhibitor Dapagliflozin in patients with CKD. J Am Soc Nephrol 2022;33:2094–107. 10.1681/ASN.2022030306 35977807 PMC9678032

[101] Heerspink HJL, Stefánsson BV, Correa-Rotter R et al. Dapagliflozin in patients with chronic kidney disease. N Engl J Med 2020;383:1436–46. 10.1056/NEJMoa2024816 32970396

[102] Cao H, Liu Y, Tian Z et al. Sodium-glucose cotransporter 2 inhibitors benefit to kidney and cardiovascular outcomes for patients with type 2 diabetes mellitus and chronic kidney disease 3b-4: a systematic review and meta-analysis of randomized clinical trials. Diabetes Res Clin Pract 2021;180:109033. 10.1016/j.diabres.2021.109033 34464675

[103] Chertow GM, Vart P, Jongs N et al. Effects of dapagliflozin in stage 4 chronic kidney disease. J Am Soc Nephrol 2021;32:2352–61. 10.1681/ASN.2021020167 34272327 PMC8729835

[104] Bakris G, Oshima M, Mahaffey KW et al. Effects of canagliflozin in patients with baseline eGFR <30 ml/min per 1.73 m^2^: subgroup analysis of the randomized CREDENCE trial. Clin J Am Soc Nephrol 2020;15:1705–14. 33214158 10.2215/CJN.10140620 PMC7769025

[105] Barreto J, Borges C, Rodrigues TB et al. Pharmacokinetic properties of dapagliflozin in hemodialysis and peritoneal dialysis patients. Clin J Am Soc Nephrol 2023;18:1051–8. 37227937 10.2215/CJN.0000000000000196 PMC10564347

[106] Barreto J, Martins M, Pascoa M et al. Dapagliflozin cardiovascular effects on end-stage kidney disease (DARE-ESKD-2) trial: rationale and design. Expert Opin Drug Saf 2024;13:1–7. 10.1080/14740338.2024.2412228 39377184

[107] Broeker A, Vossen MG, Thalhammer F et al. An integrated dialysis pharmacometric (IDP) model to evaluate the pharmacokinetics in patients undergoing renal replacement therapy. Pharm Res 2020;37:96. 32409892 10.1007/s11095-020-02832-w PMC7225193

[108] Gotta V, Dao K, Rodieux F et al. Guidance to develop individual dose recommendations for patients on chronic hemodialysis. Expert Rev Clin Pharmacol 2017;10:737–52. 28447486 10.1080/17512433.2017.1323632

[109] Heyne N. Expanded hemodialysis therapy: prescription and delivery. Contrib Nephrol 2017;191:153–7. 28910798 10.1159/000479263

[110] Canaud B, Collins A, Maddux F. The renal replacement therapy landscape in 2030: reducing the global cardiovascular burden in dialysis patients. Nephrol Dial Transplant 2020;35:II51–I7. 10.1093/ndt/gfaa005 32162663 PMC7066547

[111] Khanal A, Castelino RL, Peterson GM et al. Dose adjustment guidelines for medications in patients with renal impairment: how consistent are drug information sources? Intern Med J 2014;44:77–85. 10.1111/imj.12291 24112311

[112] Akl AI, Sobh MA, Enab YM et al. Artificial intelligence: a new approach for prescription and monitoring of hemodialysis therapy. Am J Kidney Dis 2001;38:1277–83. 10.1053/ajkd.2001.29225 11728961

[113] Barbieri C, Bolzoni E, Mari F et al. Performance of a predictive model for long-term hemoglobin response to darbepoetin and iron administration in a large cohort of hemodialysis patients. PLoS ONE 2016;11:e0148938. 10.1371/journal.pone.0148938 26939055 PMC4777424

[114] Rashidi P, Bihorac A. Artificial intelligence approaches to improve kidney care. Nat Rev Nephrol 2020;16:71–72. 10.1038/s41581-019-0243-3 31873197 PMC7591106

[115] Ghassemi M, Oakden-Rayner L, Beam AL. The false hope of current approaches to explainable artificial intelligence in health care. Lancet Digl Health 2021;3:e745–e50. 10.1016/S2589-7500(21)00208-9 34711379

[116] Baigent C, Herrington WG, Coresh J et al. Challenges in conducting clinical trials in nephrology: conclusions from a Kidney Disease—Improving Global Outcomes (KDIGO) controversies conference. Kidney Int 2017;92:297–305. 10.1016/j.kint.2017.04.019 28709600 PMC6326036

[117] Dagenais S, Russo L, Madsen A et al. Use of real-world evidence to drive drug development strategy and inform clinical trial design. Clin Pharmacol Ther 2022;111:77–89. 10.1002/cpt.2480 34839524 PMC9299990

[118] Loiseau N, Trichelair P, He M et al. External control arm analysis: an evaluation of propensity score approaches, G-computation, and doubly debiased machine learning. BMC Med Res Method 2022;22:335. 10.1186/s12874-022-01799-z PMC9795588 36577946

